# Lessons for Medical and Health Education Learned from the COVID-19 Pandemic

**DOI:** 10.3390/healthcare11131921

**Published:** 2023-07-03

**Authors:** Zhanna Gardanova, Olga Belaia, Svetlana Zuevskaya, Klavdiya Turkadze, Wadim Strielkowski

**Affiliations:** 1 Department of Psychotherapy, Pirogov Russian National Research Medical University, Ostrovitianov Str. 1, Moscow 117997, Russia; 2 Department of Infectious Diseases, I.M. Sechenov First Moscow State Medical University (Sechenov University), Trubetskaya Str. 8/2, Moscow 119991, Russiazuevskiy-alex@mail.ru (S.Z.);; 3 Department of Trade and Finance, Faculty of Economics and Management, Czech University of Life Sciences Prague, Kamýcká 129, Prague 6, 165 00 Prague, Czech Republic

**Keywords:** medical education, digital technologies, health education, anxiety, wellbeing, COVID-19 pandemic

## Abstract

Our paper analyzes lessons for medical education and health education stemming from the experience gained in the course of the COVID-19 pandemic. Moreover, it tackles the issue of the social health and psychological wellbeing of medical students involved in online education during the COVID-19 pandemic. The paper systematizes up-to-date data on how medical schools and universities have adapted to the conditions of the COVID-19 pandemic and implemented novel effective solutions for the learning process, such as transitioning from traditional in-person classes to online learning, incorporating virtual simulations and telemedicine experiences for clinical training, and collaborating with health authorities to provide support in testing and contact tracing efforts. The paper contains an analysis of various aspects of medical education, such as the changes in practical classes, the impact of the pandemic on the formation of communication skills, methods for assessing students’ knowledge and skills, and many others. It also considers case studies related to the implementation of educational programs, methodologies, and novel digital technologies in a pandemic. Additionally, the paper features an empirical study that is based on the results of our own survey that was carried out with the help of a snowball convenient sampling that involved 710 medical students between 19 and 25 years of age (56% females and 44% males) from 4 Russian regions (Moscow, Krasnodar, Kazan, and Saint Petersburg). We applied the correlation between stress scores, anxiety scores, factors of stress, and strategies for coping with stress and various economic and demographic variables (age, environment, and gender) that were analyzed using the chi-square test. Our results demonstrate that over 85% of the students in our sample yielded an above-average vulnerability to stress due to the COVID-19 restrictions. At the same time, around 61% of the students experienced severe anxiety during online education in the COVID-19 pandemic. The important factors leading to stress and anxiety were the fear of getting infected and social distancing, and the best strategy to deal with stress and increase wellbeing was self-control. Through a comprehensive review of the literature and empirical estimations, our paper identifies key areas of improvement, including curriculum adaptation, technology integration, faculty development, student support, and interprofessional collaboration. The proposed recommendations aim at strengthening medical education systems and preparing healthcare professionals to effectively navigate future pandemics.

## 1. Introduction

The COVID-19 pandemic has had a significant impact on all aspects of life, and medical and health education are no exception. Medical schools and universities had to adapt quickly to ensure the safety of students, faculty, and patients while still providing quality education under many constraints [1,2]. The pandemic has forced educational institutions in such diverse countries as the United States [3] or the Russian Federation [4] to re-evaluate their teaching methods, with many shifting to virtual platforms and solutions. This change has brought about various challenges, such as limited access to clinical experiences and hands-on training. However, it has also presented an opportunity for innovation in medical education with the introduction of new technologies and a more flexible learning environment, such as 12 practical tips based on the experience from Canada [5] or the suggestion for a mobile-learning application offering the toolkit for enhancing the managerial educational opportunities based on the Spanish experience [6].

It needs to be noted that most medical schools and universities faced the COVID-19 pandemic by offering many effective solutions for the learning process based on their experience dealing with this unprecedented situation in China [7] or Libya [8]. Their initial response to the COVID-19 pandemic was to quickly adapt to the new reality and ensure that students could continue their studies safely. This involved a shift from traditional in-person classes to online learning, which required significant changes in teaching methods and course delivery [9,10]. In addition, medical schools had to address the challenges of providing clinical training, with many hospitals restricting access due to the pandemic. Some of them implemented innovative solutions such as virtual simulations and telemedicine experiences for students, as the experience from the United States demonstrates [11]. Additionally, they worked closely with local health authorities (as shown in a case study from Germany by Said et al.) to provide support in responding to the pandemic, including contributing expertise and resources toward testing and contact tracing efforts [12]. Overall, medical schools and universities demonstrated agility in adapting to the pandemic while maintaining high standards of education for their students. The pandemic has presented a multitude of challenges for educators and students in the medical field. One of the most significant challenges has been adapting to remote learning, which has required a complete overhaul of traditional teaching methods [13,14]. At the same time, many students struggled with the lack of face-to-face interaction with peers and instructors, which can negatively impact their motivation and engagement, as another study from the Unites States demonstrates [15]. Additionally, access to technology and reliable Internet connection has been a barrier for some students. Clinical training has also been disrupted due to the pandemic, leading to delays in graduation and licensure exams [16]. Lastly, mental health concerns have emerged as a major issue among both educators and students as they navigate the stress and uncertainty caused by the pandemic while continuing to cope with the academic requirements [17,18]. The COVID-19 pandemic has forced medical schools and universities to explore innovative ways of delivering education. Remote learning technologies have become the go-to solution for institutions worldwide. One of the most significant innovations in remote learning technologies is the use of virtual classrooms [19]. Institutions have adopted video conferencing platforms such as Zoom or Google Meet to hold live lectures, discussions, and tutorials. Another innovation is the use of augmented reality (AR) and virtual reality (VR) simulations to create an immersive learning experience for students [20,21]. Additionally, institutions are using online platforms such as Coursera and edX to provide access to high-quality courses from renowned institutions worldwide. These remote teaching methods have enabled medical schools and universities to continue delivering quality education while ensuring the safety of students amidst the pandemic, as the results from many literature reviews have confirmed [22]. 

One way or another, the COVID-19 pandemic has forced medical schools and universities to adapt their teaching methods to accommodate social distancing measures. One significant challenge in this adaptation process has been maintaining clinical experience for students. To address this issue, many institutions have implemented virtual clinical rotations, allowing students to observe and participate in patient care remotely. Others have developed simulation labs that replicate real-life scenarios using mannequins and other technology [23,24]. Additionally, some institutions facilitated telemedicine visits with patients, providing students with opportunities to learn about diagnosis and treatment planning remotely. These strategies have proven effective in maintaining critical clinical experience for medical students during the pandemic (for example, using the case of the successful implementation of a new telehealth elective course at the Rutgers Robert Wood Johnson Medical School), ensuring that they are well prepared for future healthcare challenges [25]. 

Furthermore, the COVID-19 pandemic forced medical schools and universities to adapt their standardized testing and assessment procedures to ensure the safety of students and faculty [26]. One solution has been the implementation of remote proctoring, which allows students to take exams from home while being monitored through video conferencing technology. Another solution has been the use of open-book exams that assess critical thinking skills rather than memorization ability [27,28]. Many medical schools have also incorporated alternative forms of assessment, such as virtual clinical simulations, to replace in-person clinical rotations that were disrupted by the pandemic. In addition, schools have adjusted grading policies to account for the challenges faced by students during this unprecedented time [29]. These adaptations allowed medical education programs to continue providing high-quality education while prioritizing the health and safety of everyone involved. 

The COVID-19 pandemic has also taken a toll on students’ mental health and well-being, leading to anxiety, stress, and depression. In response, many institutions have implemented student support services to address mental health concerns during this challenging time [30,31]. These services include virtual counseling sessions with trained professionals, online resources for self-care and stress management, and peer support groups. Medical schools are also promoting wellness activities such as yoga sessions and mindfulness practices to help students cope with the stress of the pandemic. Such efforts have been critical in ensuring that students receive not only quality academic education but also holistic support for their overall well-being during these trying times [32]. 

Therefore, the main purpose and the focus of our paper is to draw some lessons for medical and health education from the COVID-19 pandemic. The objectives of our study were to study and summarize the up-to-date data on how medical schools and universities have adapted to the conditions of the COVID-19 pandemic. The research questions are: What experience has medical and health education derived from the pandemic and how can it be used in the future? How have the pandemic-coping strategies of medical and health schools and facilities impacted the social health and psychological wellbeing of medical students involved in online education during the COVID-19 pandemic?

**Figure 1:**
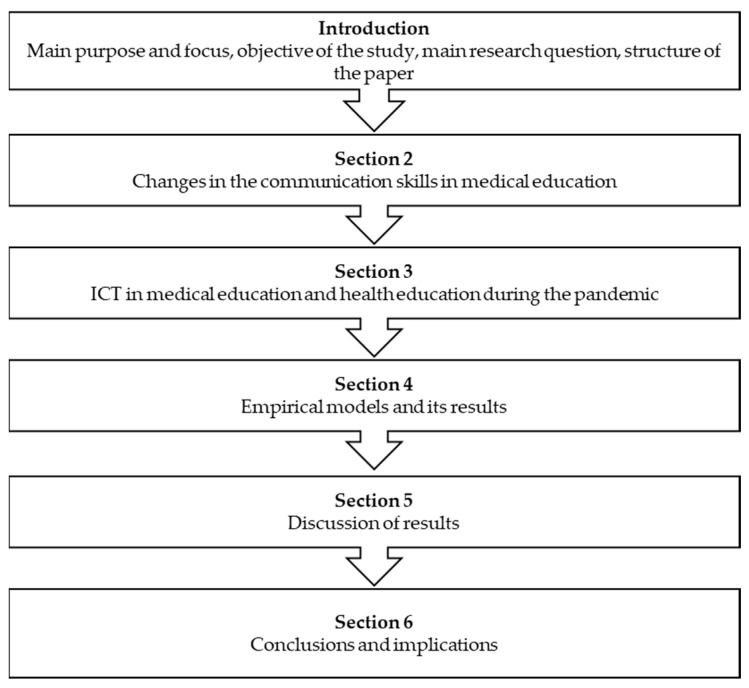
Block diagram with the brief outline of the paper’s structure and the presentation of the subsequent sections of the paper. Source: own results.

The structure of the paper is shown in the form of the block diagram in Figure 1 above (see Figure 1). The paper consists of six sections that are arranged in a logical structure with the following outline: Section 2 describes the changes in medical education. Section 3 focuses on the ICT in medical education and health education during the pandemic. Section 4 describes the survey and the data collection and presents the empirical models and its results. Section 5 discusses the obtained results. Finally, Section 6 concludes with overall conclusions, policy implications, and limitations of the study, as well as the pathways for future research.

## 2. Changes in the Communication Skills in Medical Education

The onset of the COVID-19 pandemic has brought about unprecedented changes in the way medical education is conducted. With the need for social distancing and remote learning, medical schools have had to adapt quickly to ensure that their students receive adequate training and experience [33]. The sudden shift to online learning has also impacted the formation of communication skills in medical education, which is a critical component of healthcare delivery [34]. Medical students are expected to develop strong communication skills that enable them to effectively interact with patients, colleagues, and other stakeholders in the healthcare system. However, remote learning has made it challenging for students to practice these skills in real-life situations [35]. As a result, medical schools have had to rely on virtual simulations and role-playing exercises as a substitute for clinical experience, such as the integrated virtual-reality autonomy classes described in a paper covering the results from the study with the 128 medical schools and colleges from mainland China as well as Hong Kong and Macao [36], or the blended anatomy dissection classes in the medical colleges of South Korea [37]. While these methods provide some level of training, they cannot fully replace the hands-on experience that comes with face-to-face interactions with patients and colleagues. 

**Figure 2:**
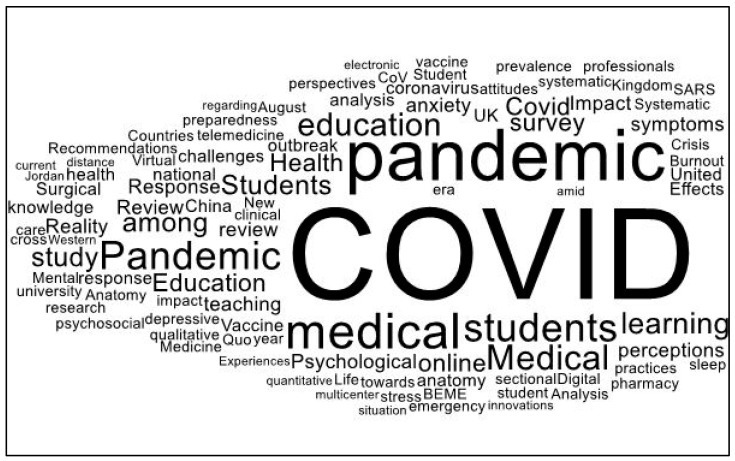
Word cloud diagram based on the titles of the 59 highly cited publications retrieved from WoS using the search terms “medical education” and “COVID-19 pandemic”. Source: own results.

Changes that occurred in medical education due to the COVID-19 pandemic became the subject of many research papers. We conducted a search of the Web of Science (WoS) Core Collection for the terms “medical education” and “COVID-19 pandemic” and obtained 4132 results from which we singled out the 59 most highly cited papers. Figure 2 that follows presents the results of the word cloud diagram based on the titles of these papers. One can notice the typical patterns with the most frequently used words presented in bold and in larger script (see Figure 2).

**Figure 3:**
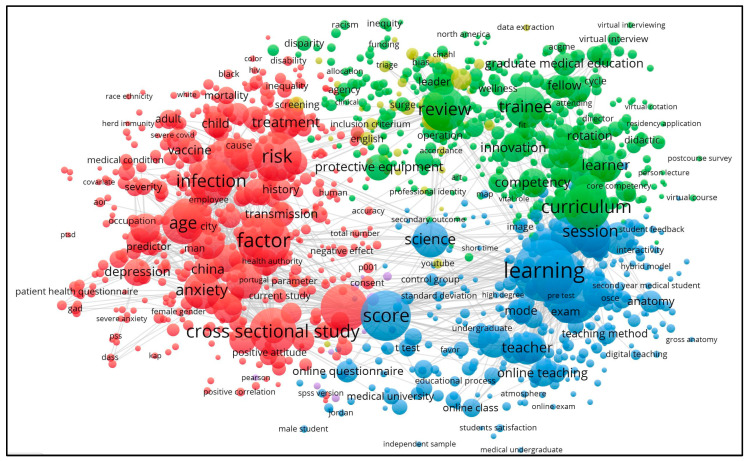
The dominant clusters of cross-sector research connected with the innovations in medical education caused by the COVID-19 pandemic. Source: own results based on VOSViewer v. 1.6.15 software (Leiden University, Leiden, Netherlands).

In addition, we conducted a bibliometric network analysis of the keywords “medical education” and “COVID-19 pandemic”. The analysis of the whole sample of 4132 publications was carried out with the help of the VOSViewer v. 1.6.15 software used for identifying the dominant trends in intersectoral research. Figure 3 below shows the results of the network map based on the text data. In total, eight main clusters were identified.

In light of this, the COVID-19 pandemic caused a significant shift to online learning in medical education. With the closure of universities and medical schools, students and educators have had to adapt to new ways of teaching and learning. The shift to online learning has affected the formation of communication skills in medical education as well [38]. Practical experience, such as hands-on clinical skills, physical examinations, and procedural training, is difficult to replicate online. Limited practical experience can have consequences for students and healthcare professionals, as it may lead to a gap in their ability to apply theoretical knowledge in real-life situations, develop critical thinking and decision-making skills, and build confidence in patient care. Additionally, the absence of direct patient interaction and exposure to diverse clinical scenarios may impact the development of communication skills, empathy, and the understanding of patient-centered care. However, with the pandemic, many clinical rotations have been canceled or postponed, depriving students of valuable hands-on experience [39]. This has forced educators to find new ways of teaching communication skills through virtual simulations and telemedicine consultations. While these methods may not completely replace real-life interactions, they can help to bridge the gap during this challenging time. It is important for educators to continue innovating and finding ways to provide quality education despite the limitations imposed by the pandemic [40]. 

Medical students all around the world have faced numerous challenges during the COVID-19 pandemic. One of the most significant hurdles was the sudden shift to online learning, which disrupted traditional classroom-based education. They had to adapt to remote learning, which has been a challenge as medical education requires hands-on experience and practical skills [41]. The pandemic also led to a decrease in clinical exposure and limited opportunities for real-life patient interaction, which is crucial for developing communication skills [42]. Furthermore, many medical students had their internships or clinical rotations postponed or cancelled due to the pandemic, resulting in a loss of valuable clinical experience [43]. The pandemic also increased stress levels among medical students as they worry about their safety and that of their loved ones while continuing their studies amidst uncertainty [44,45]. 

Overall, these challenges have posed significant obstacles for medical students’ communication skill development during the pandemic. Communication skills are critical in medical education, as they play a crucial role in the delivery of effective patient care [46]. Medical professionals must possess excellent communication skills to establish rapport with patients, elicit accurate information about their symptoms, and provide clear explanations of diagnoses and treatment options. Good communication skills also help healthcare providers to understand their patients’ concerns, beliefs, and values, which are essential for developing trust between the healthcare provider and the patient [47]. Effective communication skills also enable medical students to collaborate effectively with other healthcare professionals as part of a multidisciplinary team. They need to be able to communicate clearly with nurses, pharmacists, social workers, and other members of the healthcare team to ensure that patients receive high-quality care [48]. The COVID-19 pandemic highlighted the importance of communication skills in medical education as many aspects of patient care have been delivered remotely. 

Moreover, the pandemic drastically impacted the way that medical students are being taught [49]. One of the biggest changes is the shift toward virtual communication. With social distancing measures in place, students are now attending lectures and participating in discussions through video conferencing platforms. While this may seem like a disadvantage at first, it has brought about some positive changes in learning and skill development. Virtual communication has allowed for more flexibility and accessibility in medical education [50,51]. Students are able to attend classes from anywhere in the world and participate in discussions with their peers without having to be present in person. This has also allowed for a wider range of guest speakers to be invited to give talks or share their experiences with medical students. Additionally, virtual communication has helped to develop important skills such as effective communication over technology and remote collaboration with team members [52]. These skills are becoming increasingly important as telemedicine continues to grow and become an integral part of healthcare delivery [53]. In response to the challenges posed by the COVID-19 pandemic, educators employed various strategies to promote effective communication skills in medical education. One approach has been to incorporate technology into teaching, such as using video conferencing tools for online lectures and virtual simulations for clinical practice [54]. This not only allows students to continue learning from remote locations but also helps them to develop digital communication skills that are becoming increasingly important in healthcare. 

Another strategy has been to emphasize the importance of clear and empathetic communication when dealing with patients who may be anxious or isolated due to pandemic-related restrictions. Educators have encouraged students to listen actively, communicate clearly, and show empathy toward patients’ concerns [55]. Additionally, some institutions have provided training programs that focus specifically on communication skills in the context of COVID-19, helping students to learn how to communicate effectively with patients and colleagues while adhering to health protocols and guidelines [56]. With social distancing measures and remote learning becoming the norm, medical students have had limited opportunities for face-to-face interactions with patients and healthcare professionals. This has resulted in a lack of practical experience in communication skills such as active listening, empathetic responding, and nonverbal communication [57]. In the post-COVID-19 era, medical education will need to adapt to ensure that students develop effective communication skills. Medical schools may need to incorporate new teaching methods such as virtual reality simulations or telemedicine consultations to provide students with more opportunities for practical experience. Additionally, soft skills training may become a more integral part of the curriculum, emphasizing the importance of empathy and emotional intelligence in patient care.

To overcome the challenges of remote learning in medical education, several potential solutions can be considered. Firstly, incorporating more interactive virtual simulations and case-based scenarios can provide students with hands-on experience and decision-making opportunities. Secondly, utilizing telemedicine platforms for communication skill practice through simulated patient consultations can enhance interpersonal and patient-centered care skills. Lastly, integrating soft skills training, such as effective communication, teamwork, and resilience, into the curriculum can prepare students for the complexities of healthcare practice and promote their overall professional development.

## 3. ICT in Medical Education and Health Education during the Pandemic

The COVID-19 pandemic has had a profound impact on medical education and healthcare, necessitating a sudden shift to online learning. Traditional classroom lectures and hands-on training have been replaced by virtual classrooms and simulation-based learning as a result of the need for social distancing [58,59]. Despite the challenges associated with this transition, it has presented new opportunities for collaboration and innovation. Online learning platforms have allowed medical schools to continue educating students while prioritizing their safety. Virtual simulations have provided a secure environment for students to develop clinical skills, explore new technologies, and engage with patients remotely [60]. Furthermore, the pandemic has underscored the crucial role of information and communication technology in both medical education and healthcare delivery [61].

The benefits of using technology in medical education during a pandemic are numerous. First of all, it allows for remote learning, which is essential in times of social distancing and lockdowns. This ensures that students can continue their studies and avoid falling behind as the study from the College of Medicine at Qassim University in Saudi Arabia demonstrates [62]. Secondly, technology enables the sharing of information and resources among students and educators regardless of their location [63]. Thirdly, it allows for simulations that help students to develop practical skills without being physically present in a clinical setting [64]. Fourthly, telemedicine has become an essential tool for healthcare providers to diagnose and treat patients remotely [65]. Finally, technology provides access to online databases and journals that enable medical professionals to stay up to date with the latest research findings on COVID-19 and other diseases [66]. Overall, the use of technology has revolutionized medical education during the pandemic by providing opportunities for remote learning, practical skill development, telemedicine, and access to information resources necessary for effective healthcare provision as demonstrated by the literature reviews in the studies citied above. Thence, telemedicine has emerged as a crucial tool in the provision of healthcare services during the COVID-19 pandemic. With social distancing measures in place, telemedicine has enabled patients to consult with healthcare providers remotely, reducing the risk of infection transmission. Telemedicine can be used for a range of medical services, including consultations, diagnosis, prescription refills, and the monitoring of chronic conditions. The use of telemedicine has also helped to alleviate the burden on hospitals and clinics by reducing the number of patients physically present at these facilities [67,68]. This has allowed healthcare providers to focus on critically ill patients while still providing essential care to those who do not require hospitalization. However, telemedicine is not without its limitations.

One of the most notable innovations in medical education during the COVID-19 pandemic is the use of virtual reality (VR) training for medical professionals [69,70]. With many traditional in-person training programs and clinical rotations being suspended due to safety concerns, VR has emerged as an effective and safe alternative for teaching and practicing critical medical skills [71]. Through VR simulations, medical professionals can gain hands-on experience with procedures such as intubation, surgical techniques, and emergency response scenarios. These simulations offer a realistic environment where learners can make mistakes without risking harm to real patients. In addition to practical skills training, VR programs also offer opportunities for collaborative learning among geographically dispersed learners [72].

Thus, the use of ICTs became a must in healthcare during the COVID-19 pandemic. Nevertheless, there are still challenges and limitations that need to be addressed. One major challenge is the lack of access to technology, particularly in low-income communities and developing countries [73]. This limits the ability of healthcare providers to reach a wider population and provide essential services. To mitigate this, strategies such as providing devices and Internet access to students in need, implementing mobile-based learning platforms, and leveraging existing infrastructure such as community centers or libraries, can help to bridge the digital divide. Another challenge is the reliability and accuracy of information shared through digital platforms. Misinformation can spread quickly, leading to confusion among patients and healthcare providers [74]. Additionally, there may be concerns about data privacy and security when using online platforms for medical consultations or sharing patient information. Ensuring data privacy and security is crucial, and measures such as encrypted communication platforms, strict adherence to data protection regulations, and comprehensive training on digital ethics and security can help to alleviate concerns and safeguard sensitive patient information.

Furthermore, not all medical education programs have been able to adapt quickly enough to the shift toward digital learning. Some students may not have access to necessary equipment or Internet connections for online learning, making it difficult for them to keep up with their coursework [75]. Ensuring equitable access to technology for both educators and students has been an important aspect of utilizing information and communication technology in medical education and health during the COVID-19 pandemic. In general terms, the pandemic highlighted existing inequalities in access to technology, with students from low-income families or those living in rural areas being particularly affected. To address this issue, educational institutions have had to provide necessary technological resources such as laptops, tablets, and Internet access for both educators and students. Furthermore, there have been efforts to ensure that digital learning materials are accessible to all learners by making them available on various platforms. This includes the use of open educational resources that are free and accessible to anyone with an Internet connection [76]. For instance, telemedicine has emerged as a vital tool in providing healthcare services remotely, especially for those who are unable to visit healthcare facilities due to mobility issues or geographical barriers. The future of medical education and healthcare post-pandemic looks promising with the integration of technology. It has become clear that digital literacy and training are essential for educators and students to effectively utilize technology in medical education and healthcare. Institutions should prioritize providing comprehensive training programs that cover not only the technical aspects of using digital tools but also address critical topics such as information literacy, data privacy, and digital ethics. Offering ongoing support, resources, and mentorship can help educators and students to develop the necessary skills and confidence to navigate and leverage technology effectively, ensuring a successful integration of technology into educational and healthcare practices.

Last but not least, the pandemic highlighted the importance of technology in healthcare and medical education. Remote learning and telemedicine have become the norm, and it is expected that they will continue to be an essential part of medical education and healthcare delivery even after the pandemic [77]. Medical students can now access virtual simulations, online resources, and remote mentorship opportunities through technology. The telemedicine already mentioned above made healthcare more accessible to people who live in rural or remote areas. Patients can consult with their doctors remotely, which reduces the need for hospital visits. This not only saves time but also reduces the risk of infection transmission [78]. In conclusion, novel technologies revolutionized medical education and healthcare during the pandemic. It is expected that this trend will continue post-pandemic as technology continues to improve healthcare delivery methods and enhance medical education opportunities for students worldwide.

Therefore, the integration of technology in medical education and healthcare is poised to have a significant long-term impact on the industry. Advancements such as virtual learning platforms, telemedicine, and digital health records have the potential to revolutionize the way that healthcare is delivered, improving access, efficiency, and patient outcomes. These technologies may enable remote patient monitoring, personalized treatment plans, and enhanced collaboration among healthcare professionals. Furthermore, the increased reliance on technology is likely to drive further innovation and the development of new digital health solutions, ultimately shaping the future of the industry toward a more interconnected and patient-centric approach.

## 4. Empirical Models and Their Results

This section of our paper presents an empirical model that is based on the results of our own survey and data collection carried out with the help of a snowball convenient sampling and involving 710 respondents (medical students) between 19 and 26 years of age (56% females and 44% males) from 4 Russian regions (Moscow, Krasnodar, Kazan, and Saint Petersburg)). 

We have to acknowledge here that the snowball convenient sampling technique, while useful for reaching hidden or difficult-to-reach populations, has certain limitations. One limitation is the potential for sampling bias, as the initial participants may introduce their own biases by selectively referring individuals who share similar characteristics or perspectives. This can compromise the generalizability of the findings and introduce a lack of diversity in the sample. Nevertheless, we think that our results still represent an interesting case study and provide useful insights into the analyzed problems. We would be delighted to employ a more diverse and representative sampling strategy to enhance the generalizability of the results, but this is beyond the scope of this paper (among other things due to the financial and time constraints) and would constitute the topic of our further follow-up research.

### 4.1. Data and Survey

The data obtained for testing our empirical model were collected by the research team at the universities and medical schools in four Russian regions (Moscow, Krasnodar, Kazan, and Saint Petersburg). We employed the snowball technique and opportunity sampling to obtain a total sample of 710 valid surveys with medical and nursing students. We used an online and paper survey and ‘gatekeepers’ (local graduate students) for approaching the respondents. Due to the methodology of the sample construction described above, our sample has certain limitations that might hamper its representativeness, but it would nevertheless produce meaningful results. 

The data were collected using the online questionnaires using Google Docs from December 2021 to February 2022 and using the local ‘gatekeepers’ (a network of graduate students and research assistants who facilitated our approach to the respondents and assisted them in filling in the surveys). The reason for why we opted for this approach was that it enabled us to reach the Internet-based population, who might be quite hard to approach and reluctant to meet with the interviewers in person. 

We administered a questionnaire survey comprising 20 questions to assess stress levels. These questions covered various aspects, including identifying the most significant stressor during the pandemic (e.g., fear of the virus, vaccine, social distancing, mask wearing, online lectures, and work-related stress). We also included a question to determine the most effective stress-coping strategy (e.g., self-control, family support, colleagues, professors and friends, and spiritual support). Additionally, we used a question with five sub-categories to measure anxiety levels based on their impact on different aspects of life (e.g., labor market status, social activities, or maintaining relationships). We also collected information on living arrangements, field of specialization, and socio-demographic details (e.g., age, gender, marital status, etc.). Responses regarding anxiety were rated on a scale from 0 to 8, while responses regarding vulnerability to stress were rated on a scale from 1 (always) to 5 (never).

Table 1 that follows reports the descriptive statistics of the medical and nursing students involved in our research.

In total, 41% of the medical students were 20 years old, with over 80% of students younger than 21 years. The majority of the medical and nursing students in our sample were single and either living in student dorms or renting apartments (often sharing the rent among several tenants represented by their fellow students). More than two-thirds of the students were unemployed.

All participants were informed that the data that they provided were confidential and used for research purposes only and would not be transferred to third parties. All subjects gave their informed consent for inclusion before they participated in the study. The study was conducted in accordance with the Declaration of Helsinki, and the protocol was approved by the Ethics Committee of the Czech University of Life Sciences Prague, project No. VEV0310/2021. All the participants participated voluntarily and anonymously in the present study. All the participants signed the informed consent for participation in the study. Table 2 presents the distribution of students according to various characteristics, including stress and anxiety. 

More than 85% of the students in the sample proved to have a medium to high vulnerability to stress, while 61% of the individuals presented severe anxiety. Fear of the virus and social distancing became the most frequently named factors of stress for the students. More than half of the students in the sample considered self-control as the most important strategy for dealing with stress stemming from social distancing and online tuition in the times of the COVID-19 pandemic. Our results suggest that the effects of the pandemic were smaller between the first-year students than in the case of the senior ones, as the freshmen appeared to be less vulnerable to stress and anxiety compared to the seniors (more than 70% of the students aged 23 and older stated social distancing and online lectures as their major factors of stress). This yields a worldwide trend where the transition from in-class teaching to online learning is harder for mature students who are nearing the end of their studies.

### 4.2. Empirical Model

Table 3 below presents the results of the correlation between stress scores, anxiety scores, factors of stress, and strategies for coping with stress and various economic and demographic variables (age, environment, and gender) that were analyzed using the chi-square test.

The findings presented in Table 3 indicate that stress factors during the pandemic exhibited significant associations with students’ gender and age at a statistically significant level of 5%. Notably, a significant correlation was observed between stress-coping strategies and stress and anxiety scores, as well as age, gender, and the environment. Stress and anxiety scores displayed a strong positive correlation, while the intensity of stress showed associations with age and living conditions.

Furthermore, it was found that over 30% of 19-year-old students perceived online lectures as the most stress-inducing factor, whereas 32% of individuals aged 22 and above identified social distancing as the most challenging situation during the pandemic. Social distancing emerged as the primary concern for 28% of female students, whereas 33% of male students expressed greater fear of contracting the COVID-19 virus. Moreover, 76% of students lived alone, with the majority residing in rented accommodations (34% renting apartments and 38% staying in university and school dorms). In contrast, 18% of students lived with their parents and, among them, only 6% cohabited with other relatives. Additionally, 52% of students staying in guesthouses considered self-control as the most effective coping strategy for managing stress amid the pandemic.

Regarding age groups, stress levels, and anxiety levels, more than half of the students in each category, including 69% of females and 58% of males, reported self-control as the preferred strategy for dealing with stress. Furthermore, it is evident that students with a high or extreme susceptibility to stress also exhibited elevated levels of anxiety. For instance, 76% of students classified with high stress vulnerability experienced high anxiety, while 63% of those with an extreme stress level displayed similarly high anxiety levels.

In addition, we conducted a marginal posterior distribution for the intraclass correlation coefficient denoted by the intraclass correlation coefficient (ICC), which indicates the proportion of the variation in the scores for stress/anxiety that are caused by the between-groups heterogeneity, using a Bayesian analysis according to the respondents’ gender. The independent variables are stress and anxiety, and the dependent variables include the environment and age. The results are reported in Table 4 that follows. 

On average, the differences between males and females account for 78% of the variation in stress scores, and, for anxiety scores, this heterogeneity explains 83% of the variation. Table 4 also displays the distributions of the reliability of the random intercepts and random slope estimates across different groups. Furthermore, over 70% of the variation in anxiety scores is influenced by this heterogeneity. When considering the entire sample, factors such as age and environment significantly contribute to stress levels, while none of these factors are found to be causes of anxiety. The results reveal that labor status, environment, and age are associated with stress and anxiety (even though they might not be the causes), while gender is also a factor that might lead to anxiety.

It becomes apparent that the pandemic significantly impacted the mental health and wellness of medical students (as well as faculty, which is beyond the scope of this research but also constitutes an interesting topic of follow-up research). Medical education during the pandemic should prioritize providing support systems for those who may be struggling. This can include access to mental health resources, such as counseling services and therapy sessions. Additionally, promoting a culture of self-care and prioritizing one’s work–life balance can help to mitigate the stressors that come with medical education during any future pandemic. It is also important to create safe spaces for discussions about mental health and wellness, allowing individuals to share their experiences and seek support from their peers. Ultimately, prioritizing the mental health and wellness of medical students and faculty can lead to an improved academic performance and better patient care. Preparing for potential future pandemics or disruptions in medical education is crucial. Institutions should establish protocols for remote learning and online assessments, as well as contingency plans in case of campus closures. This includes ensuring that students have access to necessary technology and resources, such as reliable Internet connections and virtual simulation software. Faculty should also be trained in delivering effective online instruction and supporting students through virtual channels. Additionally, medical schools should consider revising their curricula to incorporate pandemic preparedness training, including infection control measures and telemedicine practices. By taking proactive steps to prepare for future disruptions, medical education can continue uninterrupted and ensure that the next generation of healthcare professionals are equipped with the skills necessary to respond to global health crises. It appears that the COVID-19 pandemic has presented significant challenges to medical education. However, it has also provided opportunities for innovation and improvement. As we move forward, it is important to continue to prioritize the safety and well-being of students and faculty while ensuring that medical education remains effective and rigorous. This can be achieved using technology, such as online learning platforms and simulation-based training. Additionally, there should be a focus on enhancing communication skills and interprofessional collaboration among healthcare professionals. Medical schools should continue to adapt their curricula to reflect changing healthcare needs in response to pandemics or other global health crises.

## 5. Discussion of Results

It appears that the COVID-19 pandemic has drastically affected all aspects of society, including medical and health education. The COVID-19 pandemic has significantly disrupted traditional teaching methods, requiring swift adjustments to ensure the safety of students, faculty, and staff while maintaining the quality of education. The necessity of physical distancing measures and restrictions on in-person gatherings forced educational institutions to quickly transition to remote learning modalities, such as online classes and virtual platforms. This shift presented various challenges, including technological barriers, limited access to resources for some students, and the need to redesign curriculums and assessments to suit the online environment. However, it also sparked innovative approaches to teaching, collaborative problem solving, and the integration of technology, paving the way for potential long-term transformations in educational practices.

Furthermore, the pandemic has forced educational institutions to adapt quickly to ensure the safety of students, faculty, and staff while continuing to provide quality education. With social distancing measures in place, traditional classroom learning has been replaced by virtual platforms and online resources. Medical schools have also had to adjust their curriculum to include new information about the virus and its impact on public health. In addition, students have been given the opportunity to contribute to the fight against COVID-19 through volunteer work and research projects. While these changes have presented challenges for educators and learners alike, they have also highlighted the importance of flexibility, innovation, and collaboration in medical education. One of the most significant changes that occurred in medical and health education due to the pandemic was the shift toward online learning and virtual simulations. With social distancing measures in place, many universities and training institutions had to quickly adapt their curriculums to an online format. This included lectures, discussions, and even clinical simulations conducted virtually. Virtual simulations allowed students to practice critical skills in a safe environment without risking exposure to the virus. Additionally, online learning provided more flexibility for students who may have been unable to attend traditional in-person classes due to travel or work restrictions. While there are challenges associated with online learning, such as limited hands-on experience and potential technology barriers, it has become an essential tool for delivering medical and health education during these unprecedented times. 

The COVID-19 pandemic necessitated a significant change in medical education, with a shift to online platforms for lectures, discussions, and clinical simulations. Online learning provided benefits such as an increased flexibility in scheduling and accessibility, enabling students to engage with educational materials from remote locations. However, challenges arose regarding the limited opportunities for hands-on experience and direct patient interactions, which are crucial for developing clinical skills and professional competencies. Institutions had to find innovative ways to simulate clinical scenarios virtually and ensure the integration of practical experiences through remote clinical rotations and telemedicine initiatives.

The COVID-19 pandemic forced medical education to adapt to new ways of learning, including the incorporation of telemedicine. Telemedicine platforms have facilitated virtual patient consultations, allowing students to observe and participate in real-time clinical interactions remotely. Additionally, telemedicine has enabled remote lectures and discussions with healthcare professionals, offering students a glimpse into the future of medicine, where technology plays an increasingly prominent role in healthcare delivery. This integration has provided valuable opportunities for students to develop skills in telehealth, patient communication, and interdisciplinary collaboration, preparing them for the evolving landscape of healthcare. The technology also allowed for remote lectures and discussions with healthcare professionals from around the world, providing access to a wider range of expertise. The incorporation of telemedicine into medical education not only enhanced the students’ understanding of virtual care but also prepared them for the future of medicine in a post-pandemic world where telehealth is likely to become more prevalent. 

The COVID-19 pandemic has highlighted the importance of interprofessional education in the healthcare sector. Interprofessional education involves collaboration among different healthcare professionals, including doctors, nurses, pharmacists, and other specialists. Collaboration among different healthcare professionals, such as physicians, nurses, pharmacists, and public health experts, has been essential in managing patient care, ensuring effective communication, and reducing transmission rates. Through interprofessional teamwork, healthcare providers have been able to develop comprehensive public health policies, implement preventive measures, and coordinate efforts to mitigate the impact of the pandemic on individuals and communities. This interdisciplinary approach has highlighted the significance of interprofessional education in fostering a holistic and collaborative healthcare system. During the pandemic, interprofessional teams have been essential in managing patient care and reducing transmission rates. They have also played a crucial role in developing and implementing public health policies to curb the spread of the virus. Interprofessional education has helped to bridge knowledge gaps between different healthcare disciplines, enabling healthcare professionals to work more effectively together. 

The pandemic has emphasized the need for ongoing interprofessional education as a means of improving collaboration among healthcare professionals and enhancing patient outcomes in times of crisis. The COVID-19 pandemic has forced medical and health education institutions to adapt their clinical rotations to ensure the safety of students and patients. In order to minimize the risk of transmission, many institutions have limited the number of students allowed on-site at a given time, resulting in reduced capacities and less issues with scheduling challenges. In response, online modules and virtual simulations have been adopted to supplement hands-on experience. Additionally, personal protective equipment (PPE) requirements, health screenings, and infection control measures have become integral parts of clinical rotations to ensure the safety of all involved. Despite these adaptations, challenges persist in providing students with adequate patient exposure and the opportunity to develop essential clinical skills. Ongoing monitoring, adherence to safety guidelines, and flexibility in curriculum delivery are essential measures to protect students and patients while maintaining the educational integrity of clinical rotations. Traditional clinical rotations, which involve hands-on learning in hospital settings, have been significantly impacted by the pandemic. Additionally, to ensure safety, institutions have implemented strict protocols for personal protective equipment, social distancing, and sanitation. Students may also be required to complete online modules or simulations before participating in clinical rotations. Additionally, institutions have had to reconsider the number of students allowed on a rotation at a given time to reduce the risk of exposure. While these changes may present challenges for medical and health education programs, they are necessary measures to protect both students and patients during this unprecedented time. 

Moreover, the COVID-19 pandemic has placed significant psychological burdens on medical students and professionals, leading to increased levels of stress, anxiety, and burnout. The disruption of routines, fear of contracting the virus, increased workload, and witnessing the toll of the pandemic on patients have contributed to these mental health challenges. In response, medical schools have recognized the importance of prioritizing mental health and have adapted their curricula accordingly. They have implemented mental health training programs, integrated self-care practices, and established support services such as counseling and peer support groups to provide resources and assistance to students and professionals in need. These measures aim to address the unique mental health needs arising from the pandemic and promote well-being within the medical community. Addressing mental health in medical education during a global crisis has become an important topic due to the recent pandemic. Medical students and professionals are facing unprecedented levels of stress, anxiety, and burnout as they work tirelessly to fight the virus. As a result, medical schools have had to adapt their curriculums to include more training on mental health and self-care practices. Many institutions have implemented virtual counseling services, mindfulness programs, and peer support groups for students. Additionally, medical schools are emphasizing the importance of self-care practices such as exercise, healthy eating habits, and regular sleep schedules to help students to manage their mental health during this challenging time. 

In addition, the recent pandemic has accelerated the integration of technology in medical education, and it is likely that this trend will continue beyond the pandemic. Technological advancements have created opportunities for collaboration among students and healthcare professionals, allowing for enhanced knowledge sharing and interdisciplinary education. Additionally, virtual platforms have improved access to education and healthcare, enabling remote learning and telehealth services, especially in underserved areas. The integration of technology in medical education has the potential to revolutionize the field by promoting efficiency, inclusivity, and innovation. With the widespread adoption of virtual learning platforms, medical students have been able to access lectures and training programs from anywhere in the world. In addition, telemedicine has become an essential tool for healthcare providers, allowing them to remotely diagnose and treat patients. This has opened new opportunities for collaboration between medical professionals across borders. As a result, medical schools are likely to incorporate more online learning into their curricula even after the pandemic subsides. This increased use of technology in medical education will not only improve access but also enhance the quality of training provided to future healthcare professionals. The COVID-19 pandemic has had a significant impact on medical and health education. The pandemic has forced educators to rethink traditional teaching methods and embrace new technologies for teaching and learning. The use of virtual simulations, telemedicine, and online learning platforms became increasingly popular during the pandemic. These changes have not only improved the quality of medical education but have also made it more accessible to learners worldwide. The pandemic has also highlighted the importance of preparing healthcare professionals for future pandemics. Medical and health education institutions must integrate pandemic preparedness training into their curricula to ensure that healthcare professionals are equipped with the necessary skills to respond effectively in times of crisis. The integration of technology, such as virtual learning platforms and telemedicine, is likely to continue as a valuable tool in medical education, promoting flexibility and accessibility. Furthermore, there is a growing recognition of the importance of incorporating pandemic preparedness training into curricula to equip future healthcare professionals with the necessary skills to respond effectively to similar crises in the future. This includes training in infection control, public health strategies, and interdisciplinary collaboration, ensuring that healthcare professionals are well prepared to navigate and mitigate the impact of future pandemics or health emergencies.

## 6. Conclusions and Implications 

### 6.1. Introductory Remarks

Overall, the COVID-19 pandemic has significantly impacted medical and health education, leading to widespread disruptions in traditional teaching methods and clinical training. Medical institutions across the globe have been forced to adapt rapidly, shifting toward such advancements as virtual learning environments, remote clinical placements, and telemedicine. The pandemic has highlighted the importance of innovative approaches to medical education that are flexible, adaptable, and resilient in the face of unforeseen challenges. The disruption caused by COVID-19 has also created an opportunity for medical educators to re-evaluate traditional teaching practices and explore new methods of delivering high-quality medical education. The COVID-19 pandemic has brought about a significant change for educators, students, and administrators alike. While this transition has allowed for the continuation of medical education during the pandemic, it has also highlighted several challenges. Medical educators must now adapt their teaching methods to suit the online environment while ensuring that students receive high-quality education and training. Virtual classrooms also require new technology and infrastructure, which may be costly for institutions. Despite these challenges, the rapid transition to remote learning has shown that it is possible to find novel solutions for providing medical and health education even during times of crisis. 

### 6.2. Summary of the Main Challenges and Innovations

The COVID-19 pandemic has posed several challenges for both students and educators in the medical field. For students, the shift to online learning has resulted in a lack of practical experience and limited hands-on training, which is crucial for their future careers. They have also faced difficulties in adapting to new technology and managing their time effectively while studying from home. Educators have had to quickly adapt to new teaching methods and technologies while ensuring that they maintain high standards of education. The pandemic has also highlighted existing disparities in access to technology and resources among students, further complicating the educational process. The pandemic has necessitated an overhaul of medical education, leading to several innovative approaches. One major innovation is the adoption of virtual learning platforms to deliver lectures and facilitate discussions. This has enabled students to continue their education remotely without compromising on the quality of instruction. Another innovation is the use of simulation software, allowing students to practice clinical skills in a safe and controlled environment. Additionally, many institutions have implemented telemedicine programs, which allow students to participate in patient consultations remotely and gain valuable experience in providing care virtually. These innovations have not only helped to maintain continuity in medical education during the pandemic but also provide a blueprint for future developments in medical education delivery. The pandemic has forced medical schools to shift toward online learning, and it has become crucial to incorporate these strategies into traditional curricula. One strategy is to use synchronous online lectures and discussions, supplemented by asynchronous content such as recorded lectures or podcasts. Another approach is to use virtual simulations or case studies that can be completed remotely. Collaborative learning can also be facilitated through online discussion forums and group projects. Additionally, the use of online assessment tools can allow for remote testing and ensure that students are keeping up with the curriculum. These strategies for incorporating online learning into traditional medical curricula will not only help students to adapt to new ways of learning but will also prepare them for future healthcare practices in a digital age. 

The COVID-19 pandemic has disrupted medical education worldwide, leading to the suspension of clinical rotations and hands-on experiences for students. However, maintaining hands-on clinical experience is crucial for the development of future healthcare professionals. It allows them to understand the practical aspects of patient care, develop critical thinking skills, and learn effective communication with patients and colleagues. In addition, hands-on experience helps students to apply theoretical knowledge in real-life situations, which leads to better patient outcomes. Thence, it is essential that medical schools find innovative ways to provide safe and structured clinical experiences for students during the pandemic while adhering to public health guidelines. This will ensure that future healthcare professionals are well prepared to meet the needs of patients in a rapidly changing healthcare environment.

### 6.3. Positive Outcomes for the Medical and Healthcare Education

It needs to be noted that, despite the challenges posed by the COVID-19 pandemic, there have been several positive outcomes and valuable lessons learned in the context of medical education. One of the most notable outcomes is that the rapid integration of technology into medical education has accelerated the adoption of innovative teaching methods and virtual learning platforms, which can enhance flexibility and accessibility for students. Furthermore, the shift to remote learning has prompted educators to explore new approaches and tools for interactive and engaging virtual teaching, promoting active learning and critical thinking skills. In addition, the pandemic has highlighted the importance of interdisciplinary collaboration and the need for healthcare professionals to adapt and work together effectively in times of crisis. Additionally, the increased emphasis on public health and infectious disease management in the curriculum has created a greater awareness of global health challenges and the importance of preparedness. Last but not least, the COVID-19 pandemic has underscored the significance of mental health support and self-care practices for both students and healthcare professionals, leading to an increased focus on promoting well-being and resilience as demonstrated in the empirical model featured in our paper. These positive outcomes and lessons learned can shape the future of medical education, fostering innovation, collaboration, and a holistic approach to healthcare delivery.

All in all, it becomes clear that the COVID-19 pandemic has unveiled the vulnerabilities and limitations of existing medical education systems when faced with global health crises. By identifying successful strategies and best practices, in the next sub-section, our paper provides actionable recommendations in various domains to strengthen medical education systems and foster adaptability in times of crisis.

### 6.4. Analysis and Recommendations

Our paper provided some analyses and recommendations stemming from the current pandemic that can be offered to improve the quality of medical and health education in the context of future pandemics. The first recommendation is the curriculum flexibility: to tackle future pandemics, the pandemic preparedness and response training need to be integrated into the curriculum, ensuring that the students are equipped with essential knowledge and skills to handle future pandemics effectively. Another key measure might be fostering flexibility in curriculum design, allowing for the rapid adaptation and incorporation of emerging research and public health guidelines during pandemics.

Another important recommendation is the focus on technology integration. Stakeholders and governments need to invest into the infrastructure and resources in order to support the seamless integration of technology into medical education, enabling virtual learning, telemedicine, and remote communication. In addition, enhancing digital literacy among faculty and students needs to be carried out by providing training and support in utilizing technology for effective teaching and learning, as well as clinical practice.

The COVID-19 pandemic demonstrated that there is also a clear need for faculty development. Faculty development programs need to be established for enhancing digital teaching skills, remote assessment techniques, and the effective use of online platforms and virtual simulations. Furthermore, faculty needs to be encouraged to engage in continuous professional development, fostering the acquisition of knowledge and skills relevant to pandemics and public health emergencies. Thence, it is recommended to establish training programs that focus on enhancing digital teaching skills, remote assessment techniques, and the effective utilization of online platforms and virtual simulations. For instance, workshops and seminars can be conducted to provide faculty with hands-on training in using educational technology tools and platforms. Additionally, encouraging faculty to engage in continuous professional development related to pandemics and public health emergencies can be beneficial. This can include participation in webinars, conferences, or courses that cover topics such as crisis management, public health protocols, and online teaching methodologies specific to healthcare education. By investing in faculty development initiatives, universities can ensure that educators are equipped with the necessary skills and knowledge to adapt to future challenges effectively.

Yet another initiative that can be recommended is student support. A comprehensive support system aimed at addressing the social, emotional, and mental health needs of students during pandemics needs to be put into place, including access to counseling services and peer support networks. Additionally, student engagement and collaboration need to be facilitated through virtual platforms, ensuring opportunities for interactive learning, virtual clinical experiences, and teamwork.

Interprofessional collaboration is another recommendation that is desirable within this context of measures. It needs to be promoted among healthcare disciplines ostering a team-based approach to pandemics and emphasizing the importance of effective communication and coordination. Moreover, partnerships between medical education institutions and public health agencies need to be encouraged and supported, facilitating collaborative efforts in preparedness, response, and research during pandemics.

### 6.5. Concluding Remarks

In summary, it can be stated that the COVID-19 pandemic clearly underscored the need to enhance the quality and adaptability of medical education systems to effectively respond to future cataclysms. By implementing the recommendations derived from the experiences of the COVID-19 pandemic, medical education institutions can better prepare students and faculty for the unique challenges presented by global health crises. A flexible curriculum, integrated technology, faculty development, robust student support, and interprofessional collaboration are all becoming crucial components for fostering resilience and ensuring the delivery of high-quality medical education in the case of any future disasters, such as possible pandemics or other catastrophes, either man-made or natural.

## Figures and Tables

**Table 1:** Descriptive statistics of the respondents.

		No. of Respondents	%
	<19 years	192	27%
	20 years	292	41%
Age	21 years	85	12%
	22 years	92	13%
	23 years	35	5%
	>24 years	14	2%
Gender	female	398	56%
	male	312	44%
Marital status	married	85	12%
	single	625	88%
	living alone	28	4%
	renting	241	34%
Living conditions	living with parents	128	18%
	living with relatives	43	6%
	living in student dorms	270	38%
	employed	483	68%
Labor market status	unemployed	227	32%
N = 710			

Source: own results.

**Table 2:** Distribution of respondents according (relative frequencies expressed in %).

Stress Category	Anxiety Category	Stress Factor	Strategy
Vulnerability to stress:	Low: 14%	Fear of vaccine: 10%	Spiritual support: 8%
Weak: 7%	Medium: 25%	Fear of virus: 33%	Self-control: 64%
Medium: 45%	Severe: 61%	Social distancing: 26%	Support of friends, colleagues, professors: 12%
High: 40%		Wearing mask: 10%	Family support: 16%
Extreme: 8%		Online lectures: 23%	
		Overworking for extra cash: 8%	

Source: own results.

**Table 3:** The correlation between stress factors and the strategy for coping with stress and anxiety.

Variable	Stress Scores	Anxiety Scores	Environment	Age	Gender
Stress factors	16.374 (0.643)	11.417 (0.642)	35.834 (0.376)	49.464 * (0.003)	14.687 * (0.062)
Stress-coping strategies	18.456 ** (0.082)	17.764 (0.043)	22.375 ** (0.097)	16.789 ** (0.095)	9.657 * (0.047)
Stress scores		39.047 * (<0.03)	19.687 ** (0.091)	27.765 * (0.004)	5.875 (0.232)
Anxiety scores	39.054 * (<0.03)		12.874 (0.423)	5.945 (0.782)	0.463 (0.988)

Note: chi-square statistics with *p*-values in brackets, * significant at 5% level, ** significant at 10% level. Source: own results.

**Table 4:** Posterior summary estimates for explaining students’ stress and anxiety scores according to gender.

Parameter	Stress					Anxiety				
	Mean	SD	25%	75%	CUSUM	Mean	SD	25%	75%	CUSUM
$\beta_{0}\left(sample\right)$	54.357	34.881	17.772	65.386	0.004	36.102	13.433	35.3776	43.476	0.502
β(sample):environment	−0.843	2.3911	−0.997	−0.997	0.064	−0.961	2.486	−1.493	−0.482	0.686
β(sample):age	0.493	2.468	0.591	0.591	0.06	−2.962	3.412	−5.497	−1.003	0.687
$\sigma_{sample}^{2}$	207.981	148.593	1.908	323.659	0	74.915	32.818	79.708	79.901	0.411
$\mu_{\beta_{0}}$	1.968	3.488	−0.602	4.361	0.601	3.649	3.488	1.491	5.785	0.702
$\mu_{environment}$	0.065	3.075	−2.066	2.306	0.537	0.096	3.096	−2.094	2.387	0.481
$\mu_{age}$	0.062	3.301	−2.086	2.477	0.536	0.453	3.098	−1.994	2.446	0.554
$\tau_{\beta_{0}}$	641.772	476.947	387.535	768.282	0.731	396.562	268.365	252.392	483.793	0.702
$\tau_{\beta_{0}:\;\beta_{environment}}$	−8.998	45.634	−28.494	12.805	0.575	−6.322	41.162	−26.623	16.478	0.479
$\tau_{\beta_{0}:\;\beta_{age}}$	−4.969	45.943	−25.989	17.645	0.346	−35.771	45.954	−54.301	−8.951	0.562
$\tau_{\beta_{0}:\;\beta_{age}}$	−18.925	47.516	−34.291	4.948	0.556	−17.930	43.417	−35.801	6.492	0.481
$\tau_{\beta_{environment}:\beta_{age}}$	0.078	2.235	−0.947	0.986	0.658	1.302	4.383	−0.901	2.898	0.571
$\tau_{\beta_{age}}$	3.605	3.234	1.749	3.276	0.644	8.734	9.601	3.457	11.481	0.591
α	2.422	2.599	0.735	2.201	0	3.781	2.894	1.802	4.821	0.634
$\beta_{0}\left(gender=male\right)$	65.302	0.000	65.303	65.303	0	41.744	1.991	39.505	43.468	0
$\beta_{0}\left(gender=female\right)$	65.296	0.000	66.302	65.302	0	42.487	0.031	42.484	42.485	0.004
$\beta_{environment}\left(gender=male\right)$	−0.997	0.000	−0.97	−0.997	0	0.407	0.706	−0.591	0.954	0
$\beta_{environment}\left(gender=female\right)$	−0.997	0.000	−0.997	−0.997	0	−1.423	0.036	−1.439	−1.439	0.006
$\beta_{age}\left(gender=male\right)$	−0.493	0.000	−0.493	−0.493	0	−0.996	0.319	−0.998	−0.944	0
$\beta_{age}\left(gender=female\right)$	−0.493	0.000	−0.493	−0.493	0	−5.463	0.097	−5.486	−5.409	0.004
$s^{2}\left(gender=male\right)$	322.567	0.000	322.567	322.567	0	76.876	4.296	73.447	79.911	0
$s^{2}\left(gender=female\right)$	322.567	0.000	322.567	322.567	0	94.865	24.359	79.722	105.988	0
ICC	0.975	0.403	0.975	0.999	0.733	0.938	0.304	0.979	0.998	0.602
$\mathrm{Reliability}\;(\beta_{R_{0}})$	0.999	0.003	0.997	1.101	0.845	0.998	0.004	0.998	0.998	0.608
$\mathrm{Reliability}\;(\beta_{R_{environment}})$	0.967	0.445	0.764	0.998	0.521	0.998	0.102	0.938	0.985	0.635
$\mathrm{Reliability}\;(\beta_{R_{age}})$	0.959	0.444	0.764	0.998	0.682	0.941	0.084	0.972	0.996	0.503
$\beta_{s^{2}}\left(acceptance\;rate\right)$	0.003	0.064	0.000	0.000	0	0.047	0.075	0.000	0.000	0

Source: own results.

## Data Availability

The data presented in this study are available on request from the corresponding author.
